# Effects of acupuncture on the hypothalamus-pituitary-adrenal axis in chronic insomnia patients: a study protocol for a randomized controlled trial

**DOI:** 10.1186/s13063-019-3964-5

**Published:** 2019-12-30

**Authors:** Chengyong Liu, Shiyu Zheng, Wenzhong Wu, Xiaoqiu Wang, Shan Qin, Yanan Zhao, Hanqing Xi, Qingyun Wan

**Affiliations:** Jiangsu Province Hospital of Chinese Medicine, Affiliated Hospital of Nanjing University of Chinese Medicine, Nanjing, Jiangsu China

**Keywords:** Acupuncture, Chronic insomnia, HPA axis, Randomized controlled trial, Study protocol

## Abstract

**Background:**

Acupuncture, as an important component of traditional Chinese medicine (TCM), has been widely applied in the treatment of chronic insomnia in China, while there is no clinical study related to its therapeutic mechanism.

**Methods/design:**

A single-center, single-blind, randomized, placebo-controlled trial will be conducted at Jiangsu Hospital of Traditional Chinese Medicine. A total of 60 patients will be registered. Eligible participants will be randomly divided into acupuncture group and sham acupuncture group (*n* = 30 cases in each group). Patients in both groups will be treated once every other day, three times per week for 4 weeks. The primary outcome measures are Pittsburgh Sleep Quality Index (PSQI) and concentrations of adrenocorticotropic hormone (ATCH), corticotrophin-releasing hormone (CRH), and cortisol (CORT). Secondary outcome measures are Insomnia Severity Index (ISI) and Fatigue Severity Scale (FSS).

**Discussion:**

This study aims to evaluate the therapeutic effects of acupuncture on chronic insomnia by using PSQI, ISI, and FSS. The mechanism of acupuncture on CIPs will be preliminarily discussed by analyzing the changes in concentrations of CRH, ACTH, and CORT before and after treatment.

**Trial registration:**

Chinese Clinical Trials Register, ChiCTR1800020298.

## Background

Insomnia is a condition of unsatisfactory sleep quality and is associated with daytime functional impairment [1, 2]. Among adults, 6–10% of patients meet the diagnostic criteria for insomnia [3]. Chronic insomnia is a severe sleep disorder. With increasing stress factors, such as working conditions and family and social pressures, the incidence of chronic insomnia is increasing. Chronic insomnia not only causes cardiovascular and psychiatric diseases reducing patients’ quality of life, but also causes high absenteeism and mortality risk, and ultimately leads to increased social burden [4–8].

Drug therapy and psychotherapy are the recommended treatments in current clinical guidelines [9–12]. Benzodiazepine receptor agonists (BZras), the most commonly used drugs for the treatment of insomnia, are effective for short-term treatment of insomnia, while their long-term therapeutic effect is limited [13], and a number of studies have reported that they may cause a great number of side effects [10, 14–19]. Due to concerns related to dependence and side effects, chronic insomnia patients (CIPs) are often eager to choose non-pharmacological treatments [20, 21]. Cognitive behavioral therapy (CBT) as a psychotherapeutic treatment for insomnia has been proven to be effective [2]. Due to lack of professionals and expensive treatments, however, CBT isn’t highly beneficial for the majority of patients [22].

Acupuncture, as an important component of traditional Chinese medicine (TCM), has been widely applied to treat a variety of diseases worldwide, especially sleep disturbances and mood disorders [23, 24]. Based on a meta-analysis, acupuncture has a superior therapeutic effect than benzodiazepines in the treatment of primary insomnia [25]. Although acupuncture is effective in the treatment of chronic insomnia, it still lacks standardized clinical studies and its therapeutic mechanism has remained elusive, restricting its clinical application.

Chronic insomnia is closely associated with an irregular sleep–wake rhythm. To explore the mechanisms of insomnia, the root causes of insomnia need to be studied further. In recent years, there have been several mainstream trends in the study of mechanisms of insomnia: dysfunction of the hypothalamus-pituitary-adrenal (HPA) axis, decline in the melatonin system function, neurotransmitter disorders, etc. [26–29].

Studies showed that stress is one of the important causes of insomnia. For individuals under chronic stress, first their amygdala will be activated, leading to activation of the HPA axis and increased secretion of CORT. Then, the concentrations of ACTH and CORT will increase, resulting in an awakening effect. Therefore, insomnia is closely associated with dysfunction of the HPA axis [30, 31]. Previous animal experiments confirmed that acupuncture is effective in regulating the level of HPA axis-related hormones [32, 33], although a limited number of clinical studies have been conducted.

## Methods/design

### Hypothesis

Based on the validity of acupuncture in treating insomnia, we hypothesize that acupuncture will improve the symptoms of CIPs, and there will be a difference between acupuncture and sham acupuncture based on the points of PSQI and concentrations of ATCH, CRH, and CORT.

### Objectives

The purpose of this study is to determine whether the mechanism of acupuncture in treating chronic insomnia is related to the HPA axis. By comparing changes in concentrations of ATCH, CRH, and CORT before and after treatment, this study is expected to verify the validity of this inference and provide a scientific basis for the therapeutic mechanism of acupuncture in treating chronic insomnia.

### Design

**Fig. 1:**
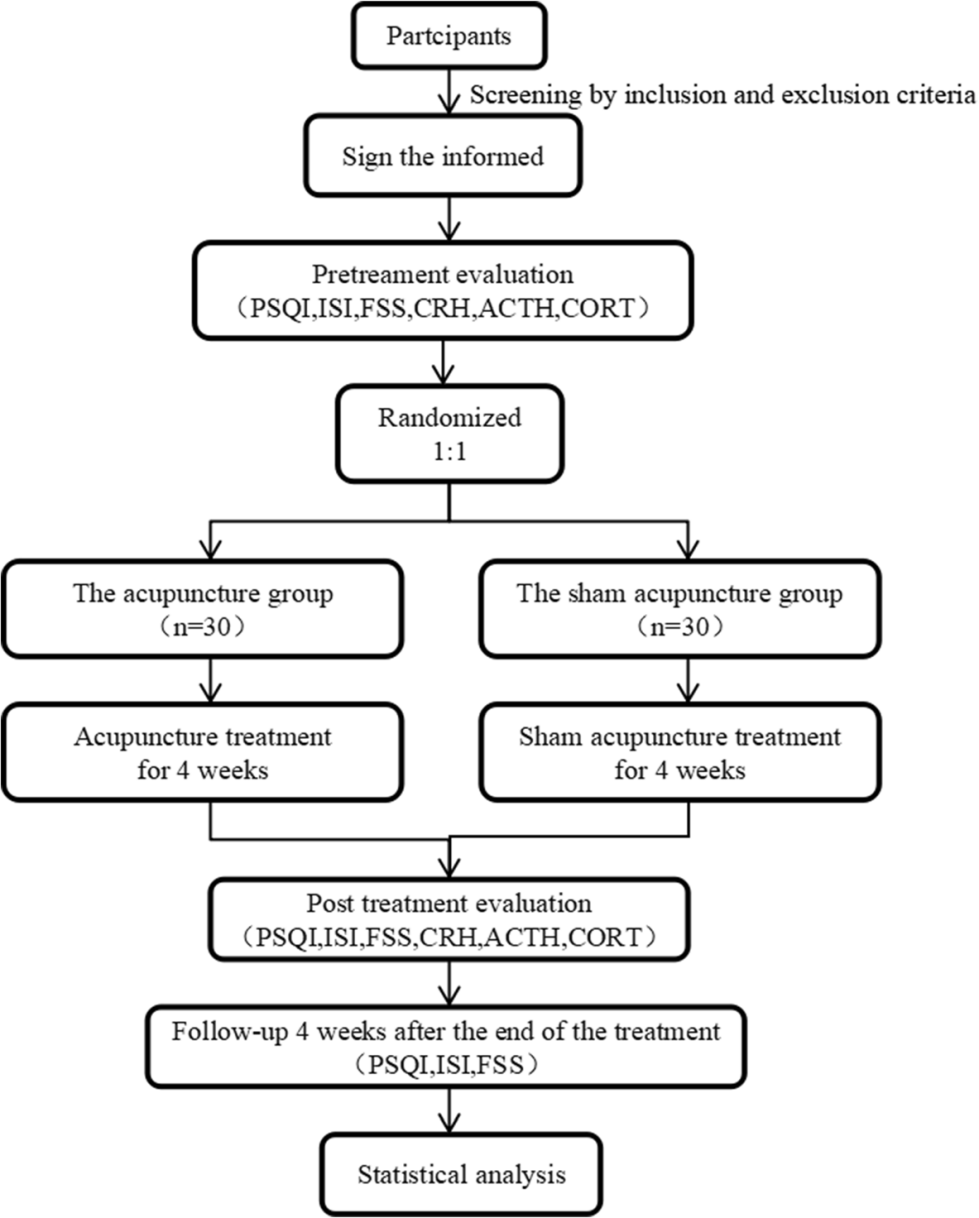
Trial procedure

In this study, 60 patients will be selected from January 2019 to December 2020 who are admitted to Jiangsu Hospital of Traditional Chinese Medicine (Nanjing, China). A single-center, single-blind, randomized, placebo-controlled trial will be conducted to compare the efficacy of acupuncture and sham acupuncture in the treatment of CIPs. Both groups will be treated once every other day, three times per week for a total of 4 weeks. The clinical trial complies with the 2010 Consolidated Standards of Reporting Trials (CONSORT) guidelines [34] as well as the Standards for Reporting Interventions in Controlled Trials of Acupuncture (STRICTA) [35]. Figure 1 shows the trial’s procedure and Table 1 details the trial’s schedule.

**Table 1:** Schedule of enrolment, interventions, and assessments

	Baseline	Intervention		P-T	F-U
	-1 week	0 day	4 weeks		8 weeks
Enrolment					
Eligibility screen	X				
Informed consent	X				
Medical history	X				
Physical examination	X			X	X
Allocation	X				
Intervention					
Acupuncture group (*n* = 30)		12 sessions of acupuncture in acupoints			
Sham acupuncture group (*n* = 30)		12 sessions of acupuncture with no skin penetration			
Outcomes					
PSQI, ISI, FSS	X			X	X
CRH, ACTH, CORT	X			X	
Others					
Success of blinding				X	
Adverse events check		X	X	X	X

*PSQI* Pittsburgh Sleep Quality Index, *ISI* Insomnia Severity Index, *FSS* Fatigue Severity Scale, *CRH* corticotropin-releasing hormone, *ACTH* adrenocorticotropic hormone, *CORT* cortisol, *P-T* post-treatment, *F-U* follow-up

### Patients

#### Recruitment strategies

There are three main approaches for recruiting patients with chronic insomnia: 1) patients admitted to the outpatient clinics of the Acupuncture and Moxibustion Department and Neurology Department of Jiangsu Traditional Chinese Medicine will be potential participants; 2) publicity brochures will be distributed in the halls of Jiangsu Hospital of Traditional Chinese Medicine to recruit potential eligible research subjects; 3) sleep-related popular science articles will be periodically published on the WeChat platform, with a brief introduction of the experimental study, to attract insomniacs to contact and participate. All participants will contact the evaluator by telephone to make an appointment for the evaluation time.

#### Inclusion criteria

Patients meeting all of the following criteria will be enrolled in the study:

1. Aged 18–65 years
2. Meeting International Classification of Sleep Disorders-Third Edition (ICSD-3) diagnostic criteria
3. Pittsburgh Sleep Quality Index (PSQI) score > 5 points
4. Not receiving psychotropic medication
5. No communication and cognitive impairment
6. Signs written informed consent form

#### Exclusion criteria

Patients meeting any of the following criteria will be excluded from the study:

1. Diseases associated with the HPA axis (pituitary tumors, adrenal hyperplasia, etc.)
2. Sleep disorders caused by an organic disease (epilepsy, diabetes mellitus, cardiovascular diseases, renal failure, etc.)
3. Sleep disorders caused by mental illness, such as depression, anxiety disorder, schizophrenia, etc.
4. Diagnosis of other sleep disorders (e.g., obstructive sleep apnea, rapid eye movement sleep behavior disorder, or restless legs syndrome)
5. Drug and alcohol addicts
6. Pregnant women, breastfeeding women, and others who are inappropriate to undergo acupuncture treatment
7. Subjects who underwent acupuncture for insomnia in the past month

### Intervention

Both groups will be treated by experienced acupuncturists with master’s degrees in medicine and doctors legally practicing medicine. All physician assistants will receive one-day professional training to familiarize them with the treatment options.

### The acupuncture group

The acupuncture points are based on *National Standard of China: Meridian Points* (GB12346–90). Participants will be in the supine position and the physician will select a 0.30 × 40 mm disposable sterile acupuncture needle produced by Suzhou Medical Products Factory Co. Ltd (Suzhou, China). The acupuncture depth is at the range of 5–10 mm after routine disinfection and acupoint selection is DU20 (Baihui), DU24 (Shenting), EX-HN3 (Yintang), HT7 (Shenmen), SP6 (Sanyinjiao). The angle and depth of needling will take the standard of each acupoint into consideration. After participants experience a needling sensation (Deqi sensation), a low-frequency pulse electro-acupuncture therapeutic apparatus (XS-998B04; Nanjing Xiaosong Medical Instrument Research Institute Co. Ltd, Nanjing, China) will be connected to the needle handle of DU20 and EX -HN3 and set to a continuous wave at a frequency of 2 Hz. The intensity of the stimulus will depend on the participant’s tolerance and stimulation will be continuous for 30 min. All acupuncture procedures will be performed by the same acupuncturist.

### The sham acupuncture group

In the sham acupuncture group, we will use a non-invasive placebo needle—a 0.30 × 25 mm blunt-headed placebo needle produced by Suzhou Huatu Medical Devices Co. Ltd. (Suzhou, China). Non-invasive placebo needles have been commonly used as placebo controls for acupuncture trials and have been recognized as a reliable sham acupuncture control tool. The acupoint selection will be the same as in the acupuncture group. A low-frequency pulse electro-acupuncture therapeutic apparatus will be connected to the needle handle of DU20 and EX -HN3, while the stimulation intensity button will not be activated. The needle will be removed after 30 min. All sham acupuncture procedures will be undertaken by the same acupuncturist.

### Outcome measures

#### Primary outcome measures

PSQI is a questionnaire for assessment of individuals’ sleep quality comprising seven parts, such as sleep quality, time to fall asleep, sleep time, sleep efficiency, sleep disorders, the taking of sleeping pills, and daytime dysfunction. The total score of PSQI is 0–21 and is inversely proportional to sleep quality; the higher the PSQI score is, the worse the sleep quality is. PSQI > 5 can be a reference threshold to judge sleep quality. In the present study, PSQI > 5 will be taken as the inclusion criterion for chronic insomnia. PSQI will be evaluated before treatment and after 4 and 8 weeks of treatment.

For determining concentrations of ACTH, CRH, and CORT, 4 ml of venous blood will be drawn and anticoagulant will be added. Plasma will be separated by centrifugation at 3000 rpm and then stored in a refrigerator (− 70 °C). A Beckman automatic chemiluminescence enzyme free analyzer (Beckman Coulter, Inc., Brea, CA, USA) will be used for the determination. Two venous blood samples from each participant will be taken at 8 am on the day before the treatment and 8 am on the day after the end of the final treatment.

#### Secondary outcome measures

Sleep severity index scale (ISI) is a self-rating scale designed by Morin and Espie (1993) [36] to evaluate the subjective feelings of individuals with insomnia. The scale includes seven items and each question has a high score of 5 points. Questions 1–3 assess the severity of insomnia and questions 4–7 assess the individual’s sleep satisfaction and daytime impact of and attention paid to insomnia. Participants will complete the questionnaire according to their sleep status in the past week. The higher the score is, the more serious the insomnia degree is. The score ranges from 0 to 28: 0–7 points indicate no problem, 8–14 points represent insomnia, 15–21 points indicate moderate insomnia, and 22–28 points demonstrate severe insomnia. Additionally, ISI will be evaluated before treatment, after 4 weeks of treatment, and after 8 weeks of treatment.

The Fatigue Severity Scale (FSS) was developed by the American Medical Association in 1989 to evaluate various chronic diseases and fatigue symptoms. This includes nine items mainly assessing the impact of fatigue on daily functions, asking about the relationship between fatigue and motivation, physical strength, working condition, family and social life, as well as scoring participants’ fatigue susceptibility and impact. The score ranges from 1 (completely disagree) to 7 (completely agree). The higher the score is, the more serious the fatigue degree is. FSS will be investigated before treatment, after 4 weeks of treatment, and after 8 weeks of treatment.

### Sample size

The calculation of sample size will be based on the change of PSQI scores. A systematic review indicated [37] that in an acupuncture group and a sham acupuncture group, a clinically significant therapeutic effect is defined as a PSQI score gap of at least 2.7 points, and we estimate that there will be 3 points in each gap between the two groups after treatment. Factors such as contamination, noncompliance, and dropout will be considered; the dropout is determined to be 15%. Therefore, each group will need approximately 30 participants to obtain statistically significant results, and we will recruit 60 participants.

### Randomization and allocation concealment

Eligible participants will be randomly assigned to the two groups in a 1:1 ratio. SPSS 22.0 software (IBM, Armonk, NY, USA) will be used to generate a random number table, which will be executed by people who have no direct contact with the participants or assessor. The random numbers will be sealed in random opaque envelopes to ensure the confidentiality of distribution.

### Blinding

Grouping results will be kept secret from participants, evaluators, and statisticians. The two groups of patients will be similar in point positions and acupuncture operation, and the patients will wear eyeshades during acupuncture to optimize the participants’ blindness. All participants will be asked to indicate whether they have received acupuncture or sham acupuncture within 5 min after treatment to assess blindness. The grouping results will not be kept secret from the acupuncturists who will provide the interventions, as they will carry out the treatment.

### Informed consent

As stated in the Declaration of Helsinki, we will inform participants about the details of our research, including objectives, characteristics, potential benefits and risks, other available treatment options, and the rights and obligations of the participants. After obtaining written informed consent, participants will be enrolled in the study. During the trial, if new ideas on research ethics emerge, the informed consent will be revised and resubmitted to the ethics committee. After approval, the informed consent will be required again. If a participant exits the trial, related data will be retained for final analysis.

### Safety monitoring

After recruitment and before randomization, all participants will undergo routine blood and liver and kidney function tests to identify and exclude patients with severe heart, liver, or kidney diseases. At the end of the study, participants will be re-examined to assess any possible side effects of the intervention. The scholars will properly address, analyze, and document adverse events (AEs) that may result from acupuncture, such as syncope, local infection, and subcutaneous hematoma. Any serious AEs associated with the trial will be immediately reported to the main researchers. The researchers will also record all other unexpected reactions as AEs, even if they are not necessarily associated with the acupuncture intervention.

### Data collection and management

The designed case report form (CRF) will collect each participant’s data and transfer them into a database based on the observed indicators. It will be kept in the archives of Jiangsu Hospital of Traditional Chinese Medicine for more than 10 years. Only members of research team will have access to the data.

### Statistical analysis

In this study, SPSS 22.0 software (IBM, Armonk, NY, USA) will be used to analyze the data. The measured data will be expressed as mean ± standard deviation, and the count data will be expressed by the ratio or composition ratio. For comparing measured data between the two groups, first normal analysis will be performed. A *t*-test will be used for the measured data conforming to normal and homogeneity of variance, and a non-parametric rank sum test will be used for non-normally distributed measured data. The data will be counted by the chi-square test or Fisher’s exact test. *P* values ≤ 0.05 will be considered statistically significant.

### Quality control

Training will be required for all participants, including acupuncturists, evaluators, and statisticians, to ensure the quality of the trials. The intervention will be based on strict adherence to standardized operating procedures. Both groups will be treated by acupuncturists with a medical master’s degree, licensed physicians, and clinical experience. All physician assistants will receive one-day professional training to familiarize them with treatment options.

To standardize clinical practices and provide clinical quality assurance, a set of clinical management practices will be developed to ensure consistency among different participants. This is helpful for management of archives, standardizing operations, as well as ensuring the feasibility and safety of the clinical research.

## Discussion

Acupuncture for the treatment of mental illness, especially insomnia, has the advantages of being low-cost, having clinical efficacy, having few side effects, and being able to be carried out in agreement with a variety of treatments. However, the mechanism of acupuncture in treating insomnia remains elusive. Therefore, we designed this single-center, single-blind, randomized, placebo-controlled clinical trial to initially validate our hypothesis. In this study, the sham acupuncture group will be used as a control group to compare the effect of acupuncture. At present, clinical research on acupuncture uses various types of sham acupuncture methods, including acupuncture on non-TCM acupoints, sham lasers on acupoints, and placebo needles [38]. In order to maximize the blinding effect of participants, this study will use a placebo needle (similar to Streitberger’s design [39]), which has a similar appearance to the needle used in the acupuncture group but has a blunt tip that does not penetrate into the skin, and both groups of participants will wear eyeshades during treatment to maximize participants’ blindness.

However, there are still some limitations in this study. 1) Two blood tests will be required before and after treatment, which will impose some difficulties on recruitment. 2) The majority of Chinese patients have some understanding of acupuncture treatment; thus, the non-invasive placebo acupuncture method in the sham acupuncture group may make the participants suspicious and interfere with treatment. For this purpose, we will require participants to wear eyeshades and train acupuncturists to answer participants’ questions during the treatment. 3) The single-center experimental design will result in a single sample, with limited representativeness, and possible experimental bias. More influencing factors should be considered and the result should be further verified and explored in a large sample population.

### Trial status

The current protocol version is 1.0 as of 6 October 2018. The randomization began (recruitment) on 1 March 2019, and 26 (43%) of 60 patients were randomized at the time of manuscript submission (1 September 2019). Recruitment is expected to end in late 2020.

## Data Availability

The full data set will be made available when this trial is completed and published.

## Abbreviations

ACTH: Adrenocorticotropic hormone
BzRAs: Benzodiazepine receptor agonists
CBT: Cognitive behavioral therapy
CIPs: Chronic insomnia patients
CORT: Cortisol
CRF: Case report form
CRH: Corticotrophin-releasing hormone
EA: Electroacupuncture
EEG: Electroencephalogram
FSS: Fatigue Severity Scale
FU: Follow-up
HPA: Hypothalamus-pituitary-adrenal
ISI: Insomnia Severity Index
PSQI: Pittsburgh Sleep Quality Index
PT: Post-treatment
RCT: Randomized controlled trial
REM: Rapid eye movement
SWS: Slow-wave sleep
TCM: Traditional Chinese medicine
